# Keratectasia severity staging and progression assessment based on the biomechanical E-staging

**DOI:** 10.1186/s40662-024-00392-3

**Published:** 2024-07-01

**Authors:** Elias Flockerzi, Berthold Seitz

**Affiliations:** Department of Ophthalmology, Saarland University Medical Center, Kirrberger Straße, Building 22, 66421 Homburg, Germany

**Keywords:** Keratoconus, Tomography, Biomechanics, ABCD staging, Corvis, E-staging

## Abstract

Until recently, corneal topography has been the gold standard in detecting keratectasia and monitoring its progression. The recently introduced ABCD tomographic keratoconus staging system focuses on anterior (“A”) and posterior (“B”) radius of curvature, thinnest corneal thickness (“C”), best-corrected visual acuity with spectacles (“D”) and is supplemented with the introduction of the biomechanical E-staging (BEST, “E”). The need for biomechanical staging arose from the fact of altered biomechanical characteristics of keratectasia in comparison to healthy corneas. Ectatic corneas usually exhibit a biomechanical weakening and greater deformation than healthy corneas when exposed to a biomechanical stressor such as a standardized air puff indentation as provided by the Corvis ST® (CST, Oculus, Wetzlar, Germany). The BEST is based on the linear term of the Corvis Biomechanical Index (CBI) and provides a biomechanical keratoconus severity staging and progression assessment within the CST software. This review traces the development of the BEST as an addition to the tomographic ABCD staging system and highlights its strengths and limitations when applied in daily practice for the detection, monitoring and progression assessment in keratectasia.

## Background

Keratoconus (KC) usually presents as a bilateral [1], quasi-inflammatory [2–5] ectatic disease of the human cornea with a prevalence estimated at 1.38:1000 according to a recently published meta-analysis [6]. When progressive, it may lead to a visual impairment because of the progressive corneal steepening and thinning. Very advanced stages may develop stromal corneal scars or even corneal hydrops. Most of the data presented in this review originate from retrospective analysis of KC patients charts from the Homburg Keratoconus Center (HKC). The HKC was founded in 2010 and comprises about 3000 KC patients in 2023. It is an observational study that aims to learn about the course of KC over lifetime and to provide an individually-adapted stage-appropriate treatment to KC patients [7].

The first presentation to the ophthalmologist is typically due to a new decrease in visual acuity in adolescence or after repeated spectacle fittings with varying conspicuous cylinder values when examined by the optician. Then, KC may be diagnosed at slit-lamp examination based on clinical signs such as Fleischer’s ring, Vogt’s striae, paracentral corneal thinning or stromal scarring. The diagnosis can also be confirmed clinically based on retinoscopy, which shows a characteristic scissoring reflex that safely diagnoses KC, but may underestimate its severity [8]. The gold standards for diagnosing KC in ophthalmological practice are corneal topography and tomography. Whilst topography may reveal an inferior corneal steepening, irregular or asymmetric bowtie-astigmatism, tomography shows corneal thinning, an apex decentration and abnormal anterior and posterior elevation data [9]. Epithelial mapping can also contribute to diagnosing KC by revealing a thinning over the area of steepening surrounded by a relatively thicker epithelium [10].

Various classification systems have been developed to stage KC, such as the Amsler-Krumeich and the topographic keratoconus classification (TKC). The Amsler-Krumeich classification includes eccentric steepening, myopia and astigmatism, mean central K readings, scarring and thinnest corneal thickness measurements. However, one eye could e.g., be classified as stage 2 KC according to the degree of myopia and astigmatism, but as stage 3 with regard to thinnest corneal thickness measurements, which is why this classification is prone to a divergence of classification results and fails to address technological advances in the field of KC management [11]. The TKC is implemented in the Pentacam® (Oculus, Wetzlar, Germany) software and provides a topographic KC staging [12].

**Fig. 1:**
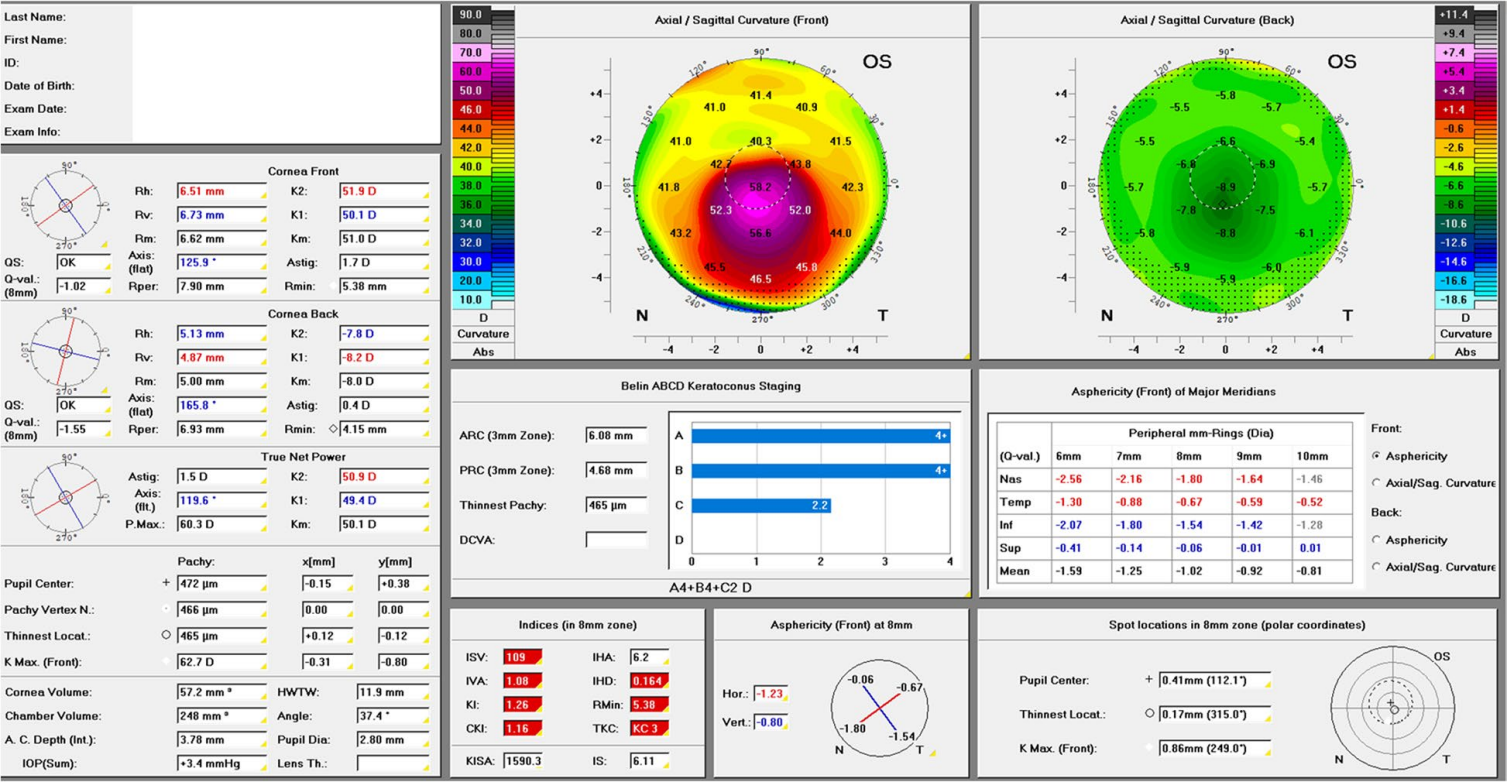
“Topometric/KC-Staging” Pentacam® display (Oculus, Wetzlar, Germany) of a left eye of a 39-year-old male keratoconus patient. Stages for “A”, “B” and “C” according to the tomographic ABCD keratoconus staging are automatically calculated and displayed under the heading “Belin ABCD Keratoconus Staging”. Staging result for this eye: A4B4C2

**Fig. 2:**
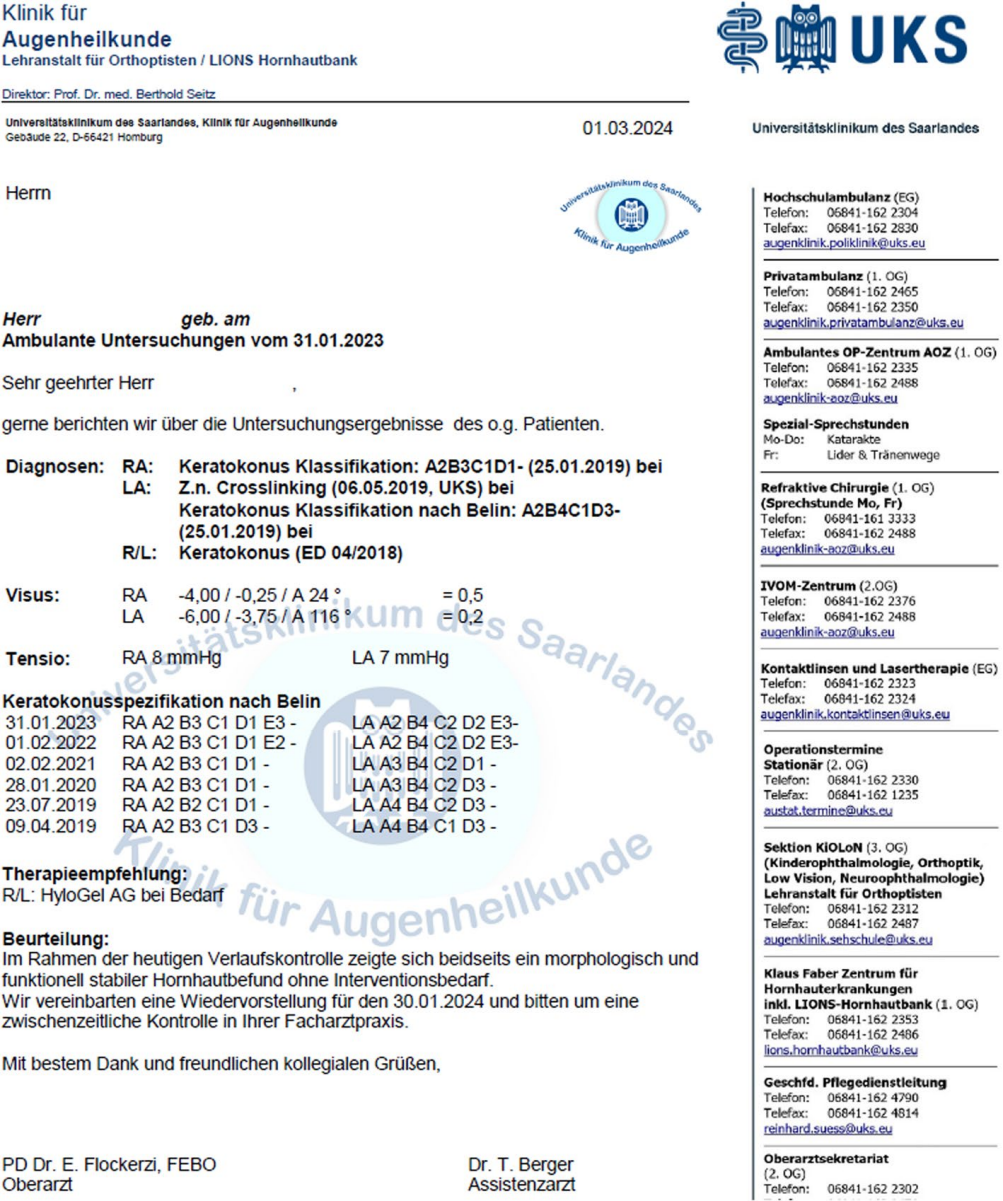
Outpatient letter to the ophthalmologist in private practice. Diagnoses including keratoconus in the right eye (“RA") and keratoconus treated with corneal crosslinking on May 6, 2019 in the left eye (“LA”). Keratonus staging over time for both eyes using the ABCDE staging system from Apr 9, 2019 to Jan 31, 2023 under the heading “Keratokonus-Klassifikation nach Belin”. Right eye (“RA”) with stable findings. Left eye (“LA”) with decreasing “A” stages after corneal crosslinking

Analysis of posterior corneal curvature, however, has gained increasing attention in recent years and is nowadays considered a screening parameter for the detection of keratectasia and a criterion that can be used to distinguish between healthy corneas, subclinical and manifested KC [13–20]. A keratectasia staging system that considers the analysis of the posterior corneal curvature is Belin and Duncan’s ABCD KC staging system (Fig. 1) that was published in 2016 and analyses anterior (“A”) and posterior (“B”) radius of curvature measured over a 3.0 mm zone centered at (“C”) the thinnest corneal thickness which also includes the patient’s best-corrected visual acuity with spectacles (“D”). More recently, the biomechanical E-staging (BEST) was given the letter “E” since it has been shown that the tomographic “A” and “B” parameters correlate significantly with corneal biomechanical indices [21, 22]. Each parameter can be classified individually in stages 0 to 4, thus resulting in a total of 625 possible tomographic combinations (A, B, C, D, with five possible numeric values for each letter) and 3125 tomographic-biomechanical combinations. When applying this staging system consistently at every follow-up examination of the patient, changes in the resulting combination of letters and numbers can give a first clue whether the patient’s KC is stable or progressive. The authors consistently use the ABCDE KC staging system in their patient charts and letters to the ophthalmologists in private practice with this intention (Fig. 2).

**Fig. 3:**
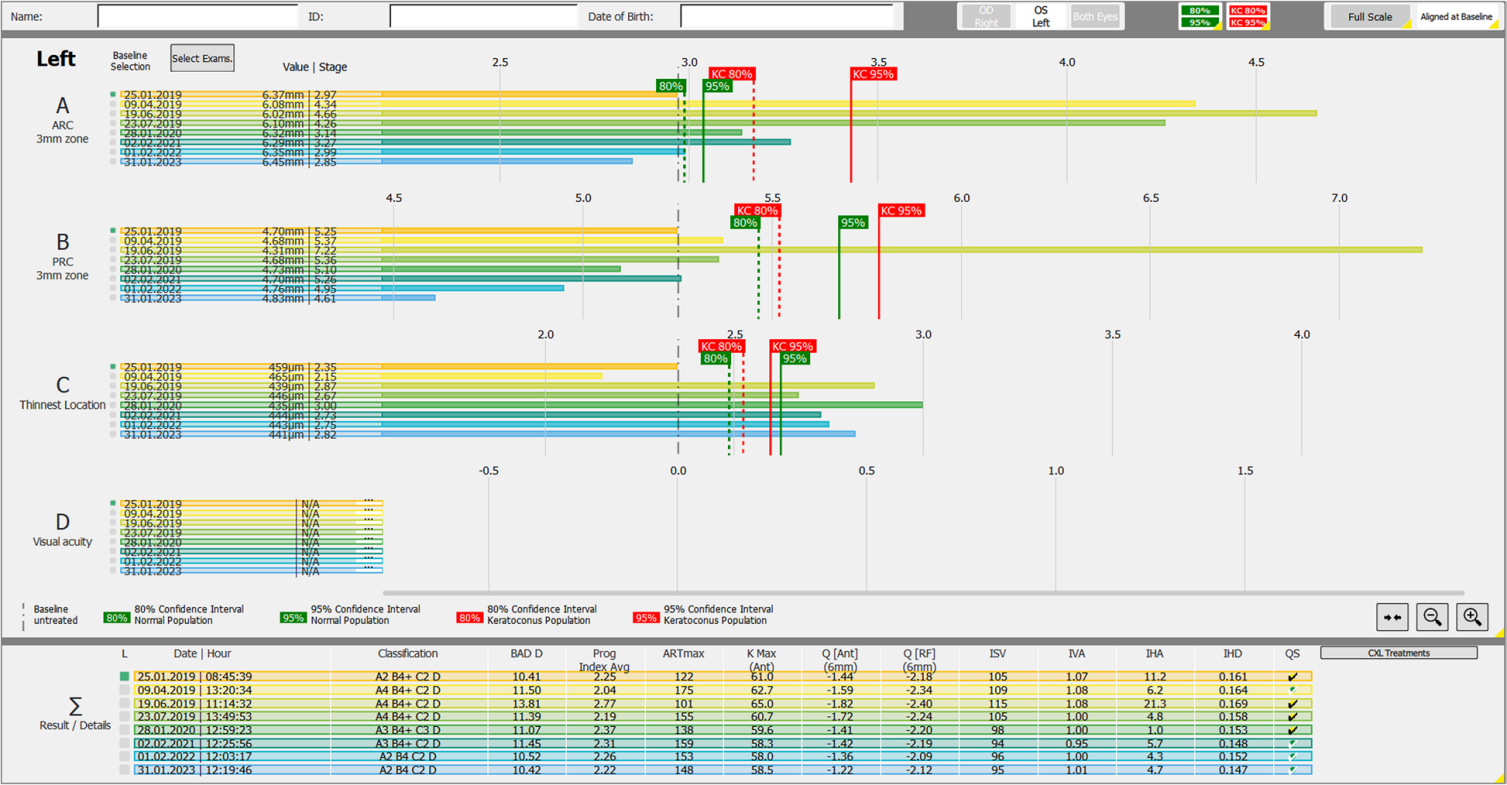
The “Belin ABCD Progression” Pentacam® display (Oculus, Wetzlar, Germany) of a left eye of a 39-year-old male keratoconus patient. Defined 80% and 95% confidence intervals in green for healthy and in red for keratoconus corneas, respectively. First follow-up examination on Apr 9, 2019 surpassing the 95% confidence interval for keratoconus. Corneal crosslinking (epithelial debridement, “accelerated protocol”, 9 mW/cm^2^, 10 min, 5.4 J/cm^2^) was performed in this eye on May 6, 2019. Pseudoprogression on Jun 19, 2019 for “A” and “B” with subsequent stabilization

More information is provided by the Belin ABCD progression display of the Pentacam® (Fig. 3), which uses a defined general level of confidence for a KC cohort. When introducing this display, the authors proposed that progression requiring corneal crosslinking for stabilization can be assumed if two of the three parameters (A, B, C) exceed the 80% confidence interval or if one of the three parameters (A, B, C) exceeds the 95% confidence interval [23]. The limitation because of a decreasing repeatability of Scheimpflug imaging with increasing KC severity [24, 25] could be overcome by a future stage-dependent evaluation of confidence intervals [26].

A baseline analysis of characteristics and application of the ABCD staging system on a large cohort of KC patients within the HKC revealed (1) more male (73%) than female (27%) KC patients, (2) more advanced posterior (“B”) than anterior (“A”) radius of curvature stages, (3) 8% to suffer from atopic dermatitis and 40% from allergies [20], (4) a mean ABCDE stage of A2B3C1D1E2 and (5) a mean Belin-Ambrósio deviation (BAD) index of 9.7 ± 8.7 among 3674 KC corneas [27].

Corneal biomechanics have been analyzed in clinical practice based on pneumotonometers that generate a standardized air puff indentation and analyze the cornea’s deformation pattern. One device that analyzes corneal biomechanics is the Ocular Response Analyzer (ORA, Reichert Instruments, Depew, USA) [28], another is the newer Corneal Visualization Scheimpflug Technology (Corvis ST®, CST, Oculus, Wetzlar, Germany). Both have been reported to distinguish between KC and healthy corneas based on a greater deformation and less corneal resistance to deformation in KC than healthy corneas. The biomechanical weakening of ectatic corneas has been reported to even precede changes on the posterior corneal curvature [29, 30], which is why biomechanical corneal analysis has gained increasing interest and importance in early KC detection.

## Main text

### Corvis Biomechanical and Tomographic Biomechanical Index

**Fig. 4:**
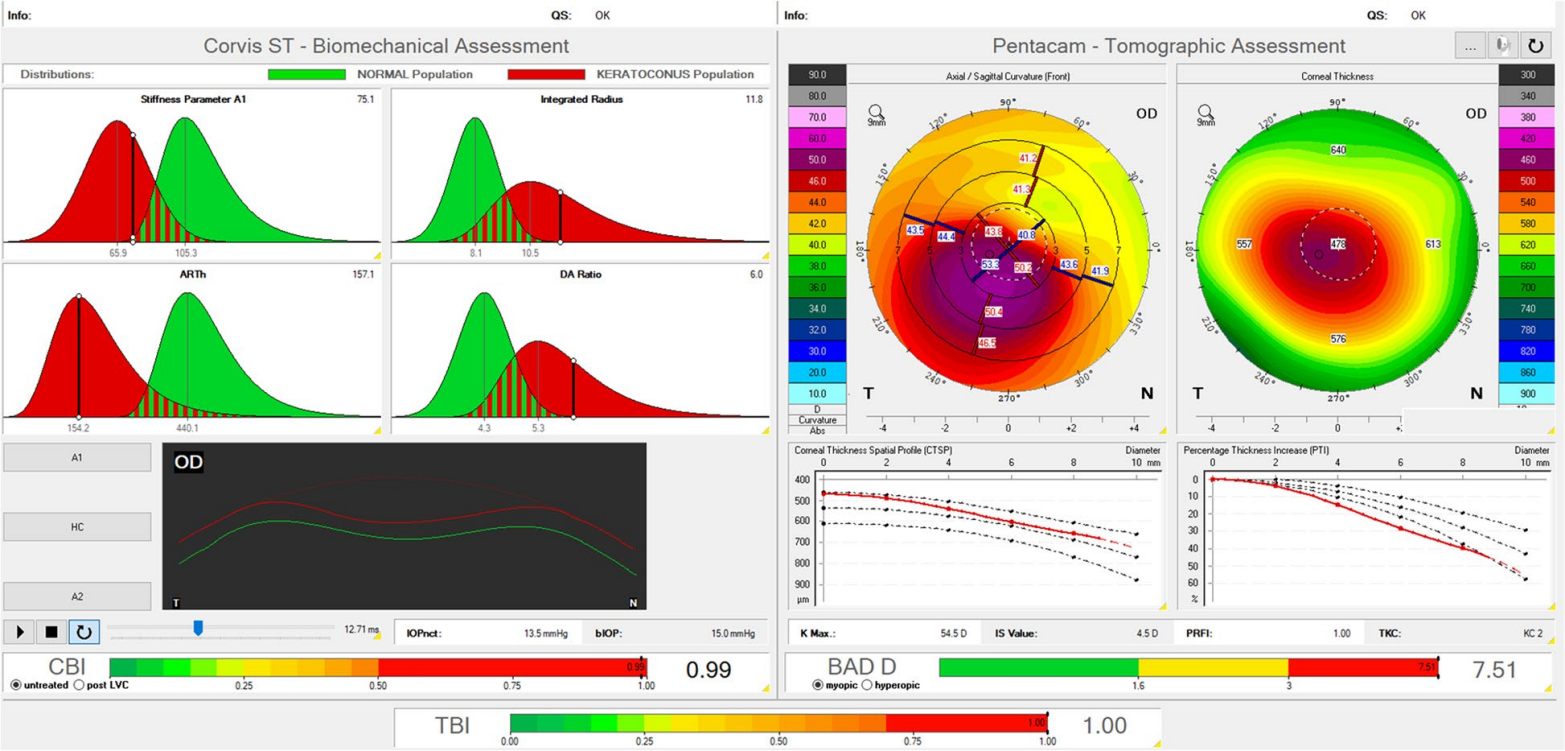
“Biomechanical/tomographic assessment” Corvis ST® (Oculus, Wetzlar, Germany) examination of a right eye of a 39-year-old male keratoconus patient. Upper left: “Corvis ST – biomechanical assessment”: individual biomechanical parameters are displayed including stiffness parameter A1 (stiffness parameter on inward applanation), integrated radius, ARTh (Ambrósio relational thickness in horizontal), and DA ratio 2 mm (deformation amplitude ratio of 2 mm). Keratoconus population highlighted in red, normal population in green. Black vertical line marking the patient's value, indicating the group the patient belongs to. Lower left: deformation curve of the cornea after application of an air puff indentation. The Corvis Biomechanical Index (CBI) makes a quasi-binary decision between a healthy cornea (green, value zero) and keratoconus (red, value one) – in this example, the value is 0.99. Right: “Pentacam – tomographic assessment ” showing anterior radius of curvature, corneal thickness, pachymetric progression, maximum keratometry value (Kmax), inferior-superior value, Pentacam random forest index (PRFI), topographic keratoconus classification (TKC) and final Belin-Ambrósio enhanced ectasia display deviation index (BAD-D). The combination of the data from the Corvis ST® and the Pentacam® in the Tomographic Biomechanical Index (TBI) is also considered quasi-binary

One of the main CST indices is the Corvis Biomechanical Index (CBI, Fig. 4), which is a logistic regression algorithm that combines several biomechanical parameters to differentiate between KC and healthy corneas [29, 31]. In a second step, the CST imports tomographic data from the Pentacam and generates the Tomographic Biomechanical Index (TBI), which combines information from the Pentacam and the CST. Both parameters behave in a quasi-binary manner. Analogous to a yes-or-no decision, the output may assume the value “0” in green for a healthy cornea and the value “1” in red for KC [31–33]. The study that introduced the CBI reported a 100% specificity and 94.1% sensitivity of the CBI in diagnosing KC within the training dataset and a 98.4% specificity and 100% sensitivity in the validation dataset [31]. The imported Pentacam display indicates topographic and tomographic KC severity based on TKC and BAD values, but from a biomechanical view, neither the CBI nor the TBI provide information about KC severity (Fig. 4). This raised the question about a biomechanical KC severity staging [21, 34–36] and one possible answer to that question is the BEST, which was developed based on the linearized CBI and thus comprises the following biomechanical parameters, that are reported to form the CBI: (1) A1 velocity (the velocity of the corneal apex at inward applanation A1), (2) deformation amplitude ratio of 2 mm (DA ratio 2 mm, the ratio between central deformation and deformation 2 mm from the corneal center), (3) integrated radius (the sum of the inverse radii of the concave state between the first and second applanation), (4) Ambrósio relational thickness in horizontal (ARTh, the ratio of corneal thickness at thinnest corneal thickness to the pachymetric index of progression) and (5) stiffness parameter A1 (SP-A1, calculated from the ratio of the force of the air applied to the corneal displacement).

### Reliability and reproducibility of the CST measurements

In our previous study, these biomechanical parameters were examined in a cohort of 173 eyes consisting of: (1) 15 healthy eyes from 15 healthy volunteers and (2) 158 KC corneas from 112 KC patients with TKC stages 1, 1–2, 2, 2–3, 3, 3–4, 4 and n = 26, 16, 36, 18, 31, 26, 5 eyes, respectively [37]. It has been reported that the reliability of successive Scheimpflug imaging and anterior segment optical coherence tomography measurements decreases with increasing KC severity [24], which led to the question whether similar observations could be found when analyzing biomechanical measurements in KC. Two studies reported that CST measurements were of excellent reliability in mild to moderate KC [38] and in three different KC severity stages [39]. To determine reliability and reproducibility at every stage of TKC, each eye was examined five times consecutively with the CST. Additionally, each eye was examined with the Pentacam prior to and after the five CST measurements with the intention to record the TKC stage and to investigate whether the repeated mechanical stress in the form of the CST measurements influenced the Pentacam measurements – and if so, to what extent. A1 velocity, DA ratio 2 mm and integrated radius increased, whereas ARTh and SP-A1 decreased with increasing KC stages. Cronbach’s alpha was calculated as a measure of internal consistency and showed a very good to excellent reliability of the measurements with values ≥ 0.834. When comparing the initial and final tomographic measurements, there was no consistent pattern of significant differences, indicating that repeated CST measurements did not affect the Pentacam’s tomographic measurement.

### Corvis Biomechanical Factor as linearized Corvis Biomechanical Index

The previous study revealed that the biomechanical parameters included within the CBI vary depending on KC stage. The first step towards a biomechanical severity staging for keratectasia was the conversion of the CBI into its linear term, which was first referred to as the “CBI beta” in 2020 [21]. The CBI beta was calculated for a representative cohort of 448 KC corneas from the HKC aiming to reflect different tomographic KC severity stages. The control group consisted of 112 healthy corneas from healthy volunteers presenting for refractive surgery evaluation [22]. A Pearson correlation between the CBI beta and the tomographic parameters (“A, B, C”) was performed and the CBI beta correlated best with “B” (posterior radius of curvature, *R*^2^ = 0.796 [22]). Due to the lack of an own scale and the increasing importance of posterior corneal curvature in keratectasia screening and diagnosis [13–19], the CBI beta was converted in a linear manner to the scale of posterior corneal curvature resulting in the Corvis Biomechanical Factor (CBiF) with the unit millimeters (CBiF = −0.24294226 × CBI beta + 6.02). The CBI beta is independent of posterior radius of curvature, the two parameters CBiF and PRC only share the same scale, but are not replaceable [22].

### The biomechanical E-staging (BEST)

In our previous study, for each of the 448 KC corneas, a CBiF value was obtained. Based on the 2.5 and 97.5 percentiles and an interval spacing of 0.58, the five E-stages were defined for the KC group [40]. The distribution of the E-stages was analyzed and found to be normal in this study group. For independent validation, the BEST was thereafter applied onto an independent data set of 860 KC corneas from study groups in Milano and Rio de Janeiro [22, 31, 41], which also revealed a normal distribution. Therefore, it could be concluded that the BEST achieved comparable and reproducible results in two different KC cohorts. It was shown that the most severe KC stage was set by parameter “B” followed by “E”, “A” and “C” [40]. As posterior elevation data and thinnest corneal thickness were diagnostic criteria in the selection of KC corneas for the study population used to create the BEST and also in Sideroudi's baseline HKC study [27], these are only frequency data and one cannot infer from this in principle that stages of “B” are generally more advanced than “E” or “A” in every KC [40]. The resulting BEST is meant to be an addition to the tomographic ABCD KC staging system that was published previously as it supplements biomechanical severity information for keratectasia. This recently introduced biomechanical staging parameter thus enables a combination of tomographic and biomechanical severity assessment in daily practice. In the following, the clinical application of the BEST shall be discussed and evaluated, whether it adds information to tomographic findings.

### The BEST-Display

**Fig. 5:**
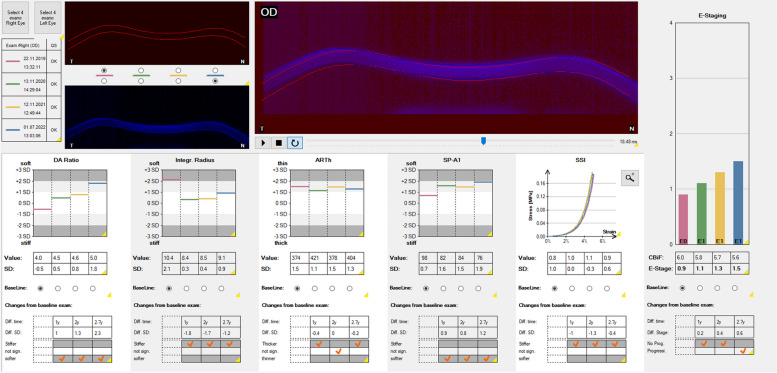
The “Homburg Biomechanical E-Staging” Corvis ST® (Oculus, Wetzlar, Germany) display of a male, 27-year-old patient with keratoconus and annual follow-up examinations from 2019 to 2022. Top left: data from the four separate examinations presented at the same time (red, green, yellow, and blue), visualization of the baseline corneal deformation (red), and corneal deformation in the final examination (blue). Upper center: superimposed display of corneal deformation visualizations. Below: absolute values and number of standard deviations from the mean value in healthy patients for (1) deformation amplitude ratio 2 mm (DA ratio), (2) integrated radius, (3) Ambrósio relational thickness in horizontal (ARTh), and (4) stiffness parameter SP-A1. (5) stress–strain index (SSI); left-shift of the curve corresponds to a higher material stiffness, a right-shift to a lower material stiffness. Right part: biomechanical E-staging for the four examination time points. Linear progression from red (Nov 22, 2019) to green (Nov 13, 2020) to yellow (Nov 12, 2021) to blue (Jul 1, 2022). Progression assessment displayed below the individual biomechanical parameters with DA ratio and SP-A1 indicating progression from the first follow-up. Progression assessment also displayed below the biomechanical E-staging indicating progression at the last follow-up examination (blue). Tomographic values for 2019, 2020, 2021, 2022: anterior simulated keratometer readings, steep meridian (K2): 44.5 D, 44.4 D, 44.4 D, 44.9 D; maximal anterior keratometry values (Kmax): 47.6 D, 46.8 D, 47.1 D, 48.4 D; thinnest corneal thickness (TCT): 525 μm, 521 μm, 513 μm, 511 μm and Belin-Ambrósio deviation (BAD) index: 3.35, 3.85, 3.58, 3.93

The BEST has been integrated into the CST software since November 2022. The “Homburg Biomechanical E-Staging” display enables the simultaneous presentation of up to four differently colored CST examinations (Fig. 5). The baseline examination is usually shown in red color, followed by up to three follow-up examinations in green, yellow and blue color. The upper left part of the chart visualizes the corneal deformation response for the first (red) and the last (blue) of the four displayed examinations and a superimposed view of these two deformation videos. The inferior part of the chart shows the absolute values and the number of standard deviations from the mean in healthy patients for the individual biomechanical parameters DA ratio 2 mm, integrated radius, ARTh, SP-A1 and stress–strain index (SSI). The SSI shows the biomechanical strength of a healthy, 50-years-old patient’s cornea in a gray colored curve (value 1.0). A SSI > 1.0 and a left shift of the curve indicates a higher corneal stiffness, a SSI < 1.0 and a right shift of the curve, a lower corneal stiffness [42]. In each follow-up examination, the CST uses the baseline examination as a reference and evaluates each parameter, whether the cornea became “stiffer”, “softer” or whether the difference was “not significant”.

On the right side, the CBiF and the resulting BEST are displayed for each of the four single examinations. While the BEST increases with increasing KC severity, it is important to note that the CBiF decreases by definition with increasing KC severity. The CBiF and BEST are therefore not individual parameters, but combine the information derived from several biomechanical parameters in a similar way to the tomographic BAD index, which is composed of several deviation values. Based on this information, the CST states below the displayed CBiF and BEST values, whether the keratectasia is progressive or not. This assessment is based on the actual values of the BEST without defined confidence intervals as opposed to the Belin ABCD progression display of the Pentacam.

### Biomechanical analysis of A0B0C0 corneas

**Fig. 6:**
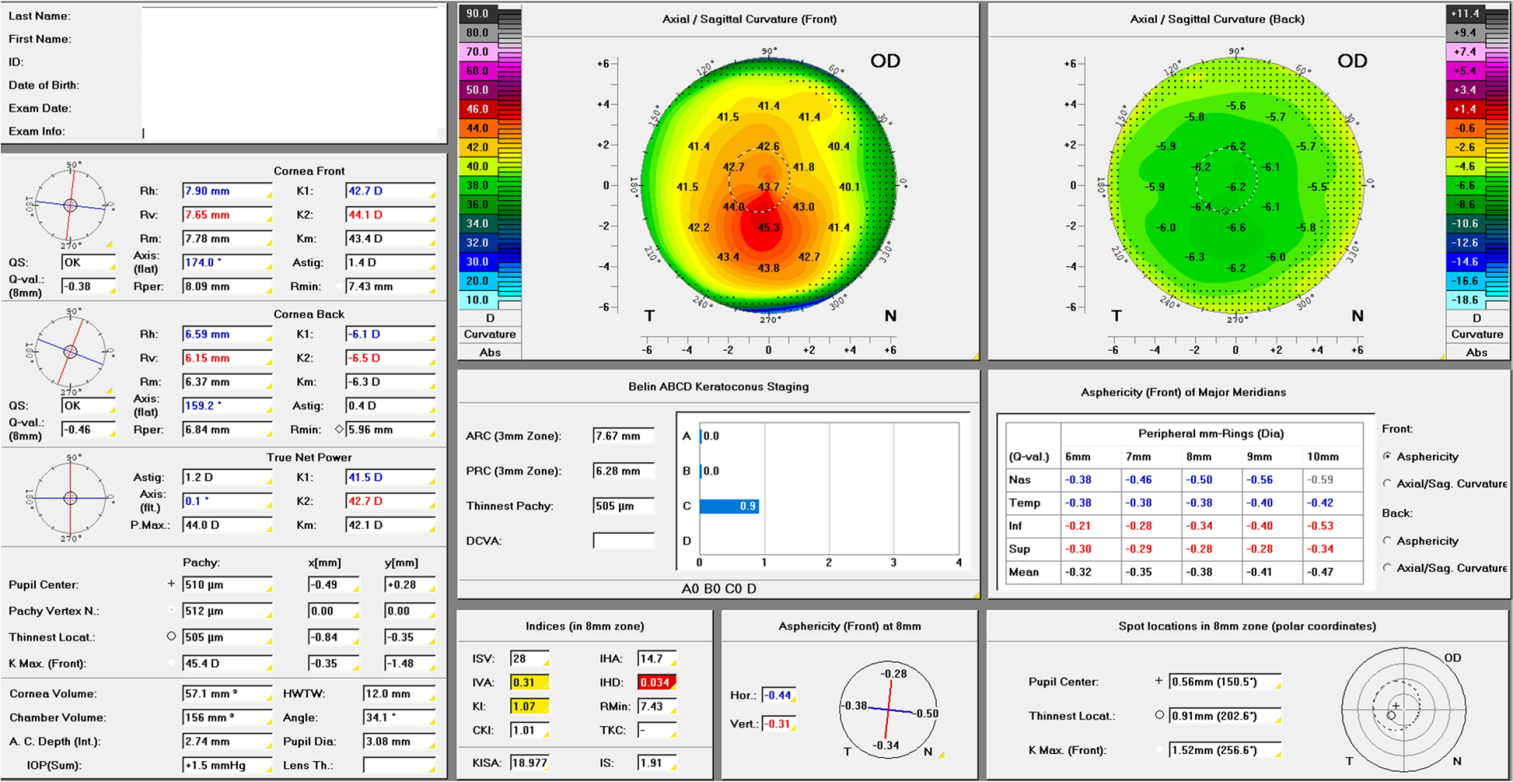
The “Topometric/KC-staging” Pentacam® display (Oculus, Wetzlar, Germany) of a 34-year-old male patient with glasses. Typical inferior steepening but resulting stage is A0B0C0. Actual values of ABC staging in the table “Belin ABCD Keratoconus Staging”: A0.0B0.0C0.9

Besides healthy corneas without ectatic regions that are classified as A0B0C0 according to the ABCD staging system, there exist also early KC stages with an A0B0C0 staging result (Fig. 6). The Pentacam automatically provides the ABC stage in the “Topometric/KC Staging” display. The example of a 34-year-old male patient wearing glasses shows an inferior steepening typical of KC, although the Pentacam staging result is A0B0C0. The table “Belin ABCD Keratoconus Staging” shows the actual “A, B, C” values together with the classification result in decimals (A0.0 B0.0 C0.9 in the example). The values are always rounded down and thus it is possible for KC corneas to be classified as A0B0C0. Consequently, A0B0C0 as a staging result does not exclude the diagnosis of KC and the ABC grading must be considered as a supplement to the ophthalmologist’s assessment of the Pentacam and not as a replacement [20]. Among the HKC patients, there were 200 (100 right and 100 left) eyes classified A0B0C0 with manifested KC in the partner eye. The mean BAD index was 1.97 ± 1.31 (mean ± standard deviation) in the A0B0C0 group and 7.62 ± 6.57 in the fellow eyes (*P* < 0.0001, Wilcoxon matched-pairs test). The mean CBI was 0.48 (A0B0C0, and thus approached 0.5 – not providing a clear decision between zero/healthy versus one/ectatic) and 0.84 (approaching one/ectatic) in the fellow eyes with manifested KC. The BEST was 0.83 (approaching stage E1) in A0B0C0 corneas and 2.20 in the fellow eyes with manifested KC, which revealed abnormal biomechanics in A0B0C0 corneas more intuitively than the CBI.

### “Unilateral keratectasia”

Sometimes, ophthalmologists may question the statement of the “Global Consensus on Keratoconus and Ectatic Diseases” [1] that unilateral KC does not exist because there exist indeed extremely asymmetrical KC cases that may show a normal tomography in one eye. Studies analyzing asymmetry in KC severity stages found that: (1) the progression of KC in one eye seems to be independent of the fellow eye [43] and that (2) among 350 KC patients, 30% had the same TKC stage in both eyes, whereas 34% showed a difference of one stage, 25% of two stages and the remaining 11% of more than two stages [44]. Another study analyzed 26 tomographically unremarkable fellow eyes of manifested KC in the other eye and found biomechanical abnormalities that helped to distinguish the tomographically unremarkable fellow eyes from healthy controls [45]. In view of these findings, it seems appropriate to carefully reflect on the definitions of the terms “subclinical KC” versus “forme fruste KC”, that have often been used interchangeably in literature. Marc Amsler examined eyes without clinical KC signs and manifested KC in the fellow eye using Placido photokeratoscopy and defined “forme fruste” as an incomplete, abortive or unusual form of a syndrome or disease [46, 47]. Consequently, a “forme fruste KC” should be a cornea with inconspicuous topographic and tomographic parameters contralateral to a cornea with manifested or advanced KC. “Subclinical KC”, however, may nowadays be diagnosed in both eyes of an individual at a very early stage and this does not necessarily mean that the disease does or will follow an asymmetrical course. One could hypothesize that the increasingly widespread use of advanced corneal tomographic and biomechanical diagnostics will achieve an earlier detection of KC than in the past, leading to increasing incidence values and earlier initiation of a stage-appropriate therapy [7].

**Fig. 7:**
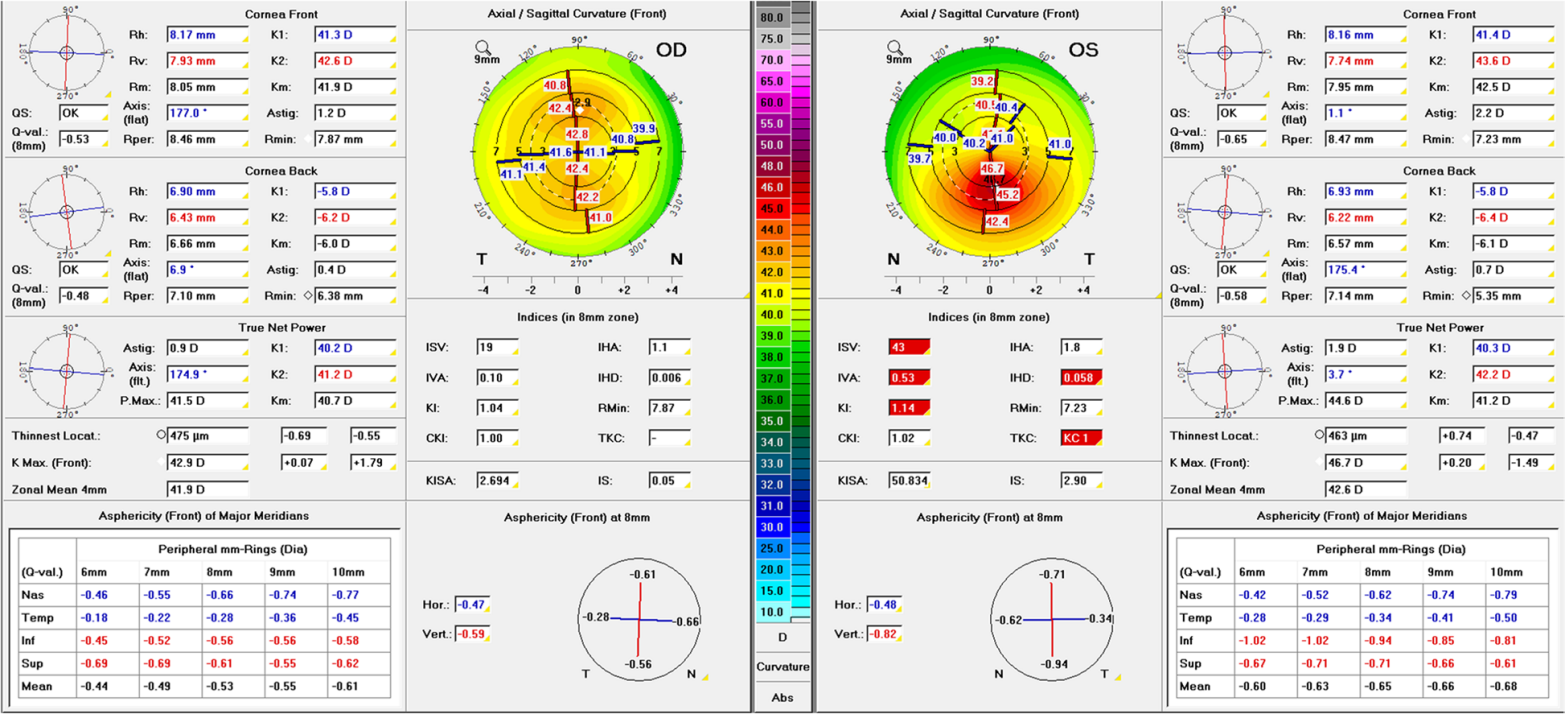
The “Topometric” Pentacam® (Oculus, Wetzlar, Germany) display of a 21-year-old male wearing glasses. Regular with-the-rule astigmatism on the right, inferior steepening on the left, with classification as keratoconus in stage TKC1

An example of a 21-year-old male patient with glasses and a bilateral visual acuity of 20/20 (Fig. 7) illustrates the importance of biomechanical analysis in “unilateral KC”. In the right eye, there is a with-the-rule-astigmatism and unremarkable KC indices, whereas the Pentacam detects TKC1 KC in the left eye. The Belin/Ambrósio enhanced ectasia display shows unremarkable elevation data for the right eye and a borderline BAD index (1.96). The left eye shows clearly abnormal findings in elevation data and BAD index (3.9). The BEST, however, displays E1.4 for the right and E1.9 for the left eye, thus revealing abnormal corneal biomechanics (E1, rounded down) in both eyes. This example highlighted that abnormal corneal biomechanics can be uncovered even in tomographically unremarkable eyes and that “unilateral KC” may be a snapshot of a highly asymmetrical keratectasia.

### Regular astigmatism or keratoconus?

**Fig. 8:**
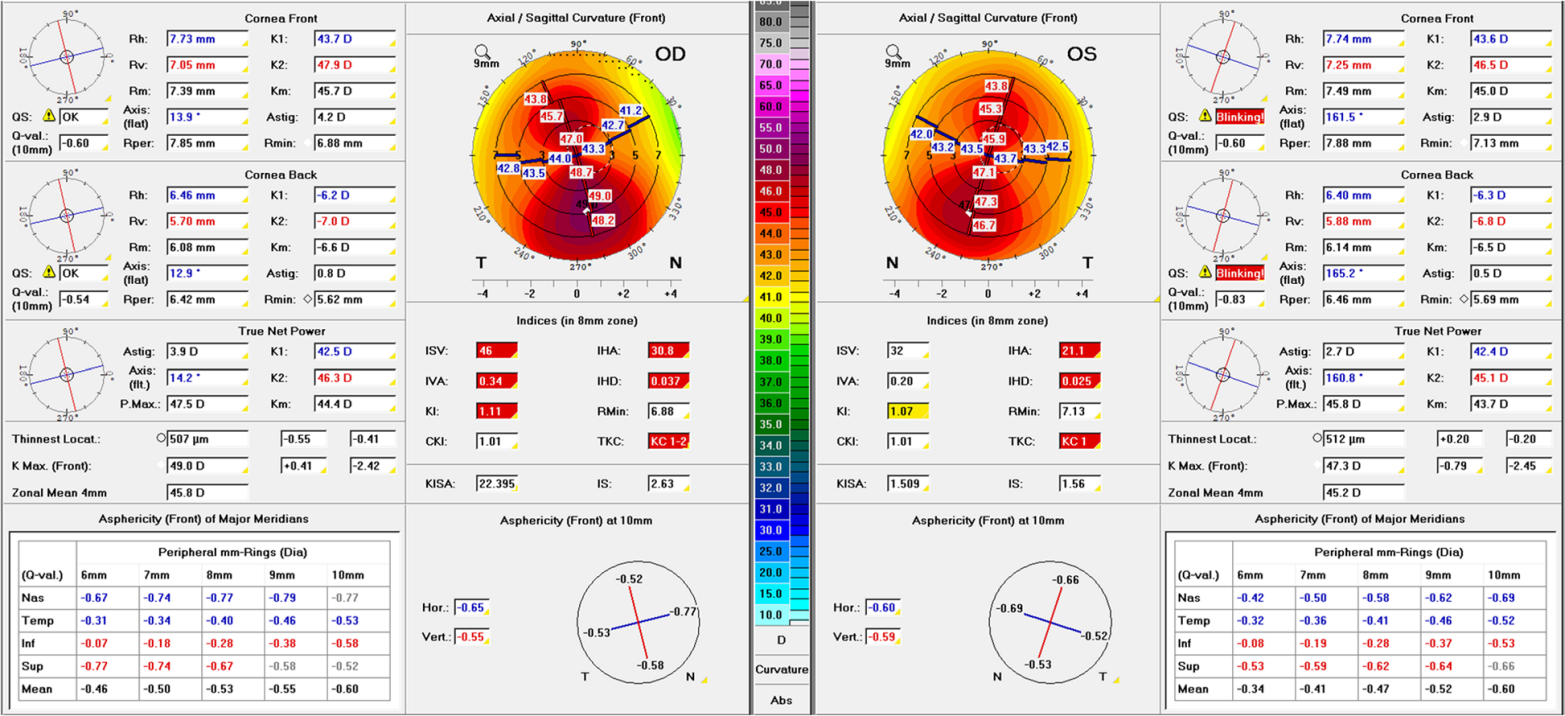
The “Topometric” Pentacam (Oculus, Wetzlar, Germany) image of a 17-year-old male wearing glasses. Regular with-the-rule astigmatism in both eyes but with distinct inferior steepening. Classification as keratoconus stage TKC1-2 on the right (Belin-Ambrósio deviation index: 2.58) and TKC1 on the left (Belin-Ambrósio deviation index: 1.56). Biomechanical E-Staging for the right eye: E1 (E1.0), and for the left eye: E1 (E1.1)

Regular astigmatism is a differential diagnosis of KC, especially in patients presenting to the ophthalmologist because of fluctuating high cylinder values. Despite thorough examination, an incipient subclinical KC cannot be ruled out with certainty in some cases. This is illustrated by the example of a 17-year-old male patient (Fig. 8) achieving a spectacle-corrected visual acuity of 20/25 with suspect of KC. The topometric Pentacam analysis showed a regular with-the-rule astigmatism in both eyes with inferior steepening and classified the right eye TKC1-2 and the left eye TKC1. The BEST showed slightly abnormal corneal biomechanics with E1.0 in the right and E1.1 in the left eye.

### BEST progression assessment

Tomographic progression assessment can be based on several parameters. An increase of the BAD index of more than 0.42 was supposed to be indicative of keratectasia progression [48]. The Save Sight Keratoconus Registry Study stated in 2021, that the Kmax increase of more than one diopter or the decrease of thinnest corneal thickness of more than 20 µm per year still remain up-to-date in daily practice [49]. A third study stated that especially in children, an initially advanced KC should be considered a risk factor for further keratectasia progression and recommended tomography changes beyond the limits of repeatability as criteria for determining progression [50]. The limits of repeatability have to be defined to determine how much of a deviation from normal indicates the change required to be considered KC progression rather than deviation owing to test variance [51]. Unfortunately, the “Global Consensus on Keratoconus and Ectatic Diseases” [1] lacked a clear definition of keratectasia progression. In Germany, the Kmax increase of more than one diopter is a legally defined progression parameter.

**Fig. 9:**
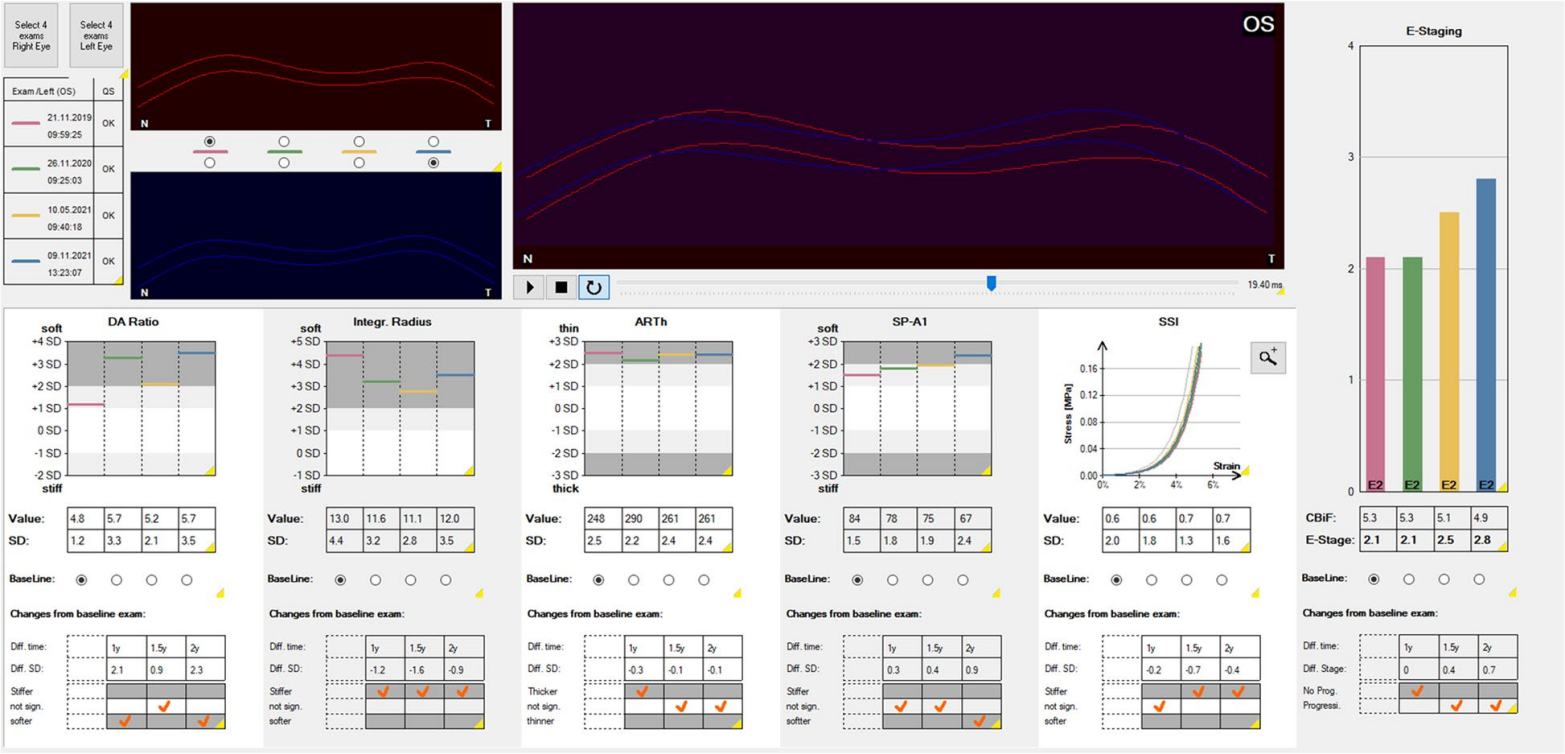
The “Homburg Biomechanical E-Staging” Corvis ST® (Oculus, Wetzlar, Germany) display of a male 24-year-old patient with keratoconus and follow-up examinations from 2019 to 2021. Red, baseline examination (Nov 21, 2019). Green, first follow-up with same biomechanical E-staging (Nov 26, 2020). Yellow, second follow-up with biomechanical progression detected by the Corvis ST® (May 10, 2021). Blue, third follow-up with further progression (Nov 11, 2021)

To evaluate how corneal biomechanics change in progressive KC corneas, a group of KC patients that underwent accelerated epithelium-off corneal crosslinking (9 mW/cm^2^, 5.4 J/cm^2^, 10 min) was examined (169 KC corneas of 169 KC patients). The baseline examination and the first follow-up examination that revealed progression, leading to the decision to perform corneal crosslinking were compared. The delta was + 1.405 for Kmax, −0.118 for “A”, −0.371 for “B”, −6.231 for “C” of the tomographic ABCD staging system, + 0.296 for DA ratio 2 mm, + 0.378 for integrated radius, −14.78 for ARTh, −2.485 for SP-A1 and + 0.2817 for BEST, which indicates, that both single biomechanical parameters and the combination of several biomechanical parameters in the BEST may also be used for keratectasia progression assessment (Fig. 9).

### BEST post-crosslinking assessment

**Fig. 10:**
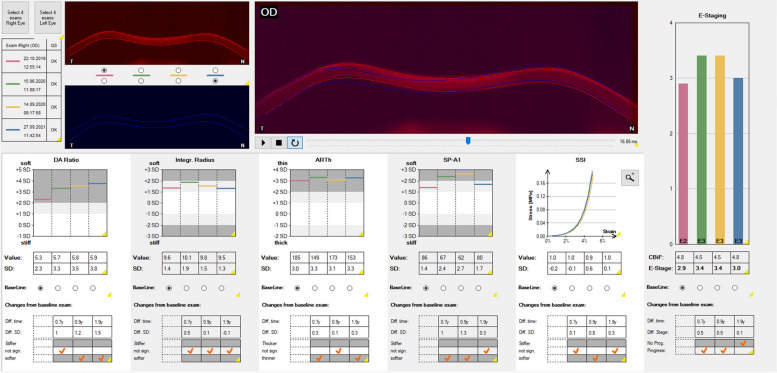
Representative course of the “Homburg Biomechanical E-Staging” after corneal crosslinking as displayed by the Corvis ST® (Oculus, Wetzlar, Germany). Female, 30-year-old patient with keratoconus and follow-up examinations from 2019 to 2021. Red, baseline examination (Oct 22, 2019). Accelerated corneal crosslinking was performed one month later (Nov 18, 2019). Green, first follow-up seven months later with biomechanical (pseudo-)progression (Jun 15, 2020). Yellow, second follow-up with same biomechanical E-staging ten months after crosslinking (Sep 14, 2020). Blue, third follow-up (Sep 27, 2021) with biomechanical stabilization near preoperative value about two years after corneal crosslinking

Corneal crosslinking is effective in halting KC progression both in adolescents [52] and adult KC patients [53]. A tomographic pseudoprogression can be observed within the first six postoperative weeks, which is not indicative of the long term effect [53]. To investigate how the BEST behaves after crosslinking, a retrospective study was performed based on 49 KC corneas of 41 patients who underwent at least one preoperative CST measurement and one postoperative measurement about one year after corneal crosslinking [54]. For some patients, there existed additional measurements at different follow-up time points, so that 22 KC corneas could be evaluated at 5 months, 49 at 11 months and 21 at 23 months after corneal crosslinking. The tomographic evaluation of Kmax and anterior corneal curvature (“A”) showed significantly lower values after crosslinking, demonstrating tomographic stabilization. The postoperative BEST value was significantly higher than preoperatively at 5 months (*P* = 0.035, paired t-test), but not significantly different at 11 months (*P* = 0.0578) and remained fairly similar to the preoperative baseline examination at 23 months (*P* = 0.9683). Therefore, the BEST cannot be used to assess progression or stability in keratectasia within the first year after corneal crosslinking, whereas it indicates stabilization at preoperative values in the long term after more than one year (Fig. 10). The answer to the question why the BEST increases within the first months after corneal crosslinking seems to lie in the Scheimpflug-based corneal thickness measurement. The majority of KC corneas (> 75%) had a postoperatively lower thinnest corneal thickness measurement and all of them showed a higher postoperative BEST value. Some KC corneas (≤ 25%) had a postoperatively higher thinnest corneal thickness measurement and showed an equal or lower BEST value. Corneal thickness influences the BEST via the biomechanical ARTh value. This raises the question, whether changed thickness values after corneal crosslinking are real or whether they represent measurement artifacts. Some studies reported an underestimation of corneal thickness after corneal crosslinking by Scheimpflug cameras [55, 56] because of stromal hyperdensities within the first year after crosslinking, causing a pachymetric artifact. Consequently, the postoperative BEST value increase could be interpreted as another artifact related to that Scheimpflug camera-related pachymetric artifact. One could assume based on these results that it requires the human cornea one to two years to approach its preoperative biomechanical status after corneal crosslinking. The postoperative stiffening effect of corneal crosslinking could be observed earlier. Individual biomechanical parameters such as the integrated radius and SSI indicated an increase in corneal stiffness as early as one month after corneal crosslinking [57, 58].

### BEST assessment after intracorneal ring segment implantation

Intracorneal ring segments were initially developed for the correction of myopia and later on used to improve visual acuity in advanced KC corneas delaying the need of keratoplasty. A recent meta-analysis reported that the femtosecond-laser assisted intracorneal ring segment implantation is effective in achieving good visual, keratometric and refractive results [59], especially in KC patients with unsuccessful contact lens fitting and initially poor visual acuity results. A reliability study revealed a reliable assessment of corneal refractive power in KC after intracorneal ring segment implantation with both Scheimpflug-imaging and anterior segment optical coherence tomography [60], which indicates that the quality and reliability of Scheimpflug-imaging, as used also by the CST, is most likely to be not impaired by and after intracorneal ring segment implantation.

**Fig. 11:**
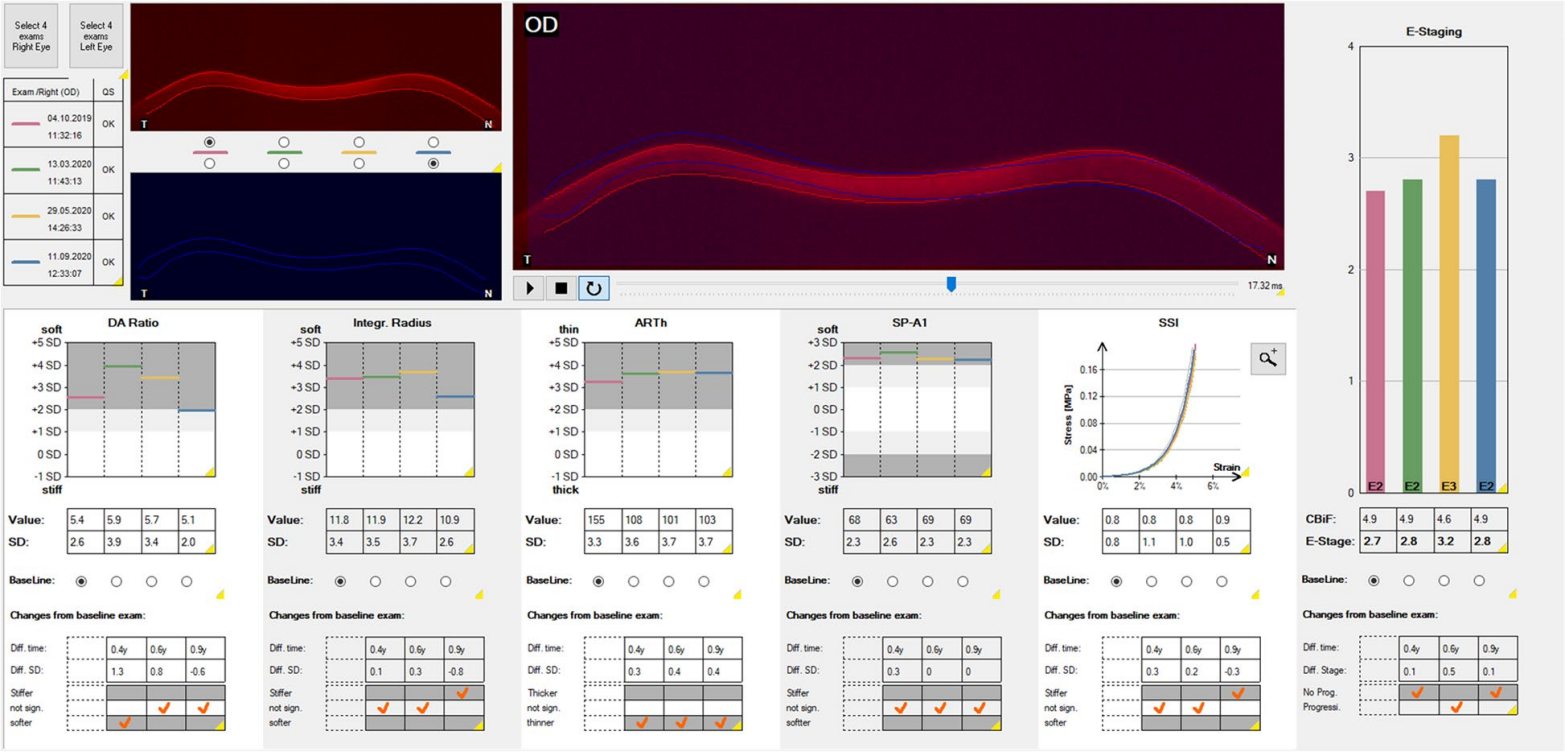
Representative course of the “Homburg Biomechanical E-Staging” after implantation of intracorneal ring segments as displayed by the Corvis ST® (Oculus, Wetzlar, Germany). Female, 39-year-old patient with keratoconus and follow-up examinations from 2019 to 2020. Red, baseline examination (Oct 04, 2019). Implantation of Intacs® SK intracorneal ring segments was performed (Nov 28, 2019). Green, first follow-up with slightly higher biomechanical E-staging about four months later (Mar 13, 2020). Yellow, second follow-up with markedly higher biomechanical E-staging (May 29, 2020). Blue, third follow-up (Sep 11, 2020) with decreasing biomechanical E-staging comparable to baseline about ten months after intracorneal ring segment implantation

Furthermore, for this treatment option, 49 KC corneas treated with intracorneal ring segment implantation were retrospectively evaluated if they had at least one preoperative and one postoperative CST measurement about one year after surgery. Similar to the post-crosslinking evaluation, there existed additional measurements at different follow-up time points for some eyes. There were significantly lower Kmax and “A” values postoperatively when compared to preoperatively. In comparison to preoperative values, ARTh was the only biomechanical parameter that showed consistently significantly lower values postoperatively. Lower ARTh values are indicative of thinned central cornea with a steep increase of corneal thickness towards the periphery [31]. As the thinnest corneal thickness measurement did not change postoperatively, it can be assumed that the ring segments implanted in the 6 mm zone are the actual cause of the steeper thickness increase towards the corneal periphery. This results in a relatively thinner central corneal thickness when compared to the implantation zone. Since these thickness changes in the midperiphery of the cornea persist as long as the ring segments stay in place, and because ARTh influences the BEST, it can be assumed that this leads to a permanent influence on the BEST, that showed higher values than preoperatively in all follow-up examinations (Fig. 11). Consequently, despite not affecting purely biomechanical measurements in early follow-up examinations [61], the implantation of intracorneal ring segments may be detected by the CST and the parameters ARTh and BEST.

### BEST assessment for other keratectasia

The CBiF has been developed for and validated based upon two KC cohorts [22, 40]. It is based upon the CBI, which was developed to detect KC and to distinguish between healthy and KC corneas [31]. Its use should therefore be restricted to the detection, staging, monitoring and progression assessment of ectatic corneal diseases [62]. A use for the detection of primary angle-closure glaucoma [63] must be critically questioned also in view of the fact that different dynamic responses of the cornea have been described in high tension glaucoma when compared to normal tension glaucoma [64].

However, it is of interest, how the BEST behaves in keratectasias other than KC. Therefore, topographic and biomechanical data of 75 corneas diagnosed with pellucid marginal degeneration (PMD) and 75 age- and gender-matched healthy controls were investigated [65]. The diagnosis of PMD is difficult and subject to controversies, as the diagnosis has often been based on topographic patterns such as a “kissing birds”, “crab claw” or “lobster claw”. Despite showing corneal curvature anomalies, these patterns ignore the actually proving criterion of an inferior limbus parallel thinning in PMD, leading to an overdiagnosis of PMD cases in the past [66, 67] because many of these cases might have been inferior KC instead of PMD. The arcuate thinning band characteristic of PMD is typically located in a distance of 1 to 2 mm from the limbus which is often not covered by the topographic imaging showing anomalies as described above. Therefore, PMD should only be diagnosed based on a full corneal pachymetry map covering at least 12 mm in diameter [66]. The biomechanical analysis of 75 PMD corneas and 75 healthy controls revealed a positive correlation between the BEST and K1, K2, Kmax, BAD index and a negative correlation with central corneal thickness, indicating that topographic and biomechanical impairments do correlate also in PMD. However, one has to keep in mind that the CST measures the center of the cornea, whereas the pathology in PMD is located rather in the paralimbal region. Although a hinge-effect between the peripheral limbal and (para-) central cornea separated by the arcuate thinning band 1 to 2 mm from the limbus in PMD can be expected to influence corneal biomechanics of the central cornea, an ideal biomechanical analysis in true PMD cases would also measure the cornea's periphery.

### Challenges and limitations

The CST measurements are based on a surface-based central corneal deformation. Although being a non-contact examination which hardly represents impairment for the patient, it has some limitations. First, it does not assess local depth-dependent biomechanical properties. Second, the sclera, intraocular pressure, extraocular tissues and other factors such as the ocular pulse amplitude also contribute to the corneal deformation behavior [68]. Different approaches have been used to overcome these limitations. Brillouin spectroscopy offers a spatial resolution at different points of the cornea with ectatic areas in KC showing a significantly smaller Brillouin frequency shift than central normal corneas [69]. Optical coherence elastography measures a series of images while mechanically applying an applanation-like perturbation, which provides a characterization of spatial depth-dependent biomechanical properties and alterations within the corneal stroma [70].

This led to the finding that KC corneas show a selective weakening of the anterior corneal stroma [71]. Furthermore, the one-time deformation-based analysis of corneal biomechanics does not consider the ocular pulse amplitude [72]. The pulsatile ocular blood flow causes changes of the intraocular volume, thereby resulting in a fluctuation of intraocular pressure of 3 mmHg on average in humans [73]. This leads to an expansion of the cornea along the curvature and compression through the thickness depending on the intraocular pressure and the ocular pulse amplitude [73]. Ocular pulse elastography, however, enables spatiotemporal quantifications of corneal displacements and strains in response to the ocular pulse [73].

Furthermore, the BEST analysis after corneal crosslinking raised the question, whether it should be expected to increase or to decrease after corneal crosslinking. Since a higher BEST indicates biomechanical destabilization or progression, one would expect it to decrease after corneal crosslinking because of the stiffening effect. However, corneas with a postoperatively lower thinnest corneal thickness measurement showed higher BEST values than preoperatively, whereas corneas with a postoperatively higher corneal thickness measurement showed an equal or lower BEST value [54]. This is due to the inclusion of ARTh in the CBI (and CBiF) algorithm, with lower ARTh values resulting in higher BEST values. ARTh results out of the division between the thinnest corneal thickness and the pachymetric progression index with lower values indicating a thinner cornea and / or a faster thickness increase towards the corneal periphery [31]. This in turn raises these questions: (1) whether thinnest corneal thickness really decreases after corneal crosslinking [53], (2) if so, when does it return to preoperative values and (3) whether ARTh-based corneal thickness data should be included in the BEST. If assuming that the apex location is shifted more along the vertical than on the horizontal meridian, there could be a difference in BEST along the horizontal and vertical meridians if considering ARTh (horizontal) for the horizontal and ARTv (vertical) for the vertical meridian. However, the current CST is not able to measure ARTv (personal communication with Dr. Sven Reisdorf, Oculus, Wetzlar, Germany).

The inclusion of a thickness-based parameter in the CBI algorithm has already been discussed in literature and an adjusted CBI without including ARTh (aCBI) has been proposed in the past [74]. When applying the CBI and the aCBI to the original CBI study dataset, the CBI with ARTh included showed superiority in terms of sensitivity and specificity [75]. Thus, ARTh remained included in the CBI algorithm as both a correction parameter for thickness and an independent separating parameter [75]. Despite being based on the same parameters, the CBI and the BEST have different aims: While the CBI as a logistic regression analysis aims to distinguish between normal and keratoconus corneas at first glance based on one single measurement, the BEST as a linear parameter has been created for staging and progression analysis. In this view and based on the BEST results after corneal crosslinking or the implantation of intracorneal ring segments, it could make sense indeed to question, whether the BEST should include a corneal thickness parameter or whether it should be limited to purely biomechanical parameters.

## Conclusions

Keratoconus is biomechanically characterized by an abnormal deformation behavior when exposed to a standardized air puff indentation. The biomechanical weakening increases with increasing KC severity and can be measured with dynamic corneal response parameters. The initial CST indices, CBI and TBI, distinguish in a quasi-binary manner between KC and healthy corneas. The BEST adds the cornerstone of corneal biomechanics to the existing tomographic ABCD KC staging system and is based upon the linearized CBI. It is integrated into the CST software in the “Homburg Biomechanical E-Staging” display. The BEST reveals abnormal biomechanical characteristics in A0B0C0 corneas, which may be diagnosed as (1) “forme fruste KC” if there is manifested KC in the fellow eye or as (2) “subclinical KC” if there are unremarkable tomographic findings with abnormal biomechanics in both eyes. Tomographically progressive KC corneas also showed a biomechanical progression in dynamic corneal response parameters. The BEST display provides a biomechanical progression assessment for up to four single CST examinations that can be displayed at same time. After corneal crosslinking, the BEST value returns to baseline values after more than one year and is thereafter useful for the assessment of the crosslinking effect in the long term. After implantation of intracorneal ring segments, the BEST value remains permanently slightly higher than preoperatively because of the thickness increase in the midperipheral implantation area. The CST and BEST detect biomechanical abnormalities in PMD that correlate with topographic severity, indicating that the BEST may also be suited for the assessment of other corneal ectasia than KC, which offers an intuitive addition to the tomographic staging system for use in daily practice as the ABCDE KC staging.

## Data Availability

The datasets used and/or analyzed during the current study are available from the corresponding author on reasonable request.
